# Chromosomal identification of cryptic species sharing their DNA barcodes: *Polyommatus (Agrodiaetus) antidolus* and *P. (A.) morgani* in Iran (Lepidoptera, Lycaenidae)

**DOI:** 10.3897/CompCytogen.v11i4.20876

**Published:** 2017-11-21

**Authors:** Vladimir A. Lukhtanov, Nazar A. Shapoval

**Affiliations:** 1 Department of Karyosystematics, Zoological Institute of Russian Academy of Sciences, Universitetskaya nab. 1, St. Petersburg 199034, Russia; 2 Department of Entomology, Faculty of Biology, St. Petersburg State University, Universitetskaya nab. 7/9, St. Petersburg 199034, Russia

**Keywords:** Ancestral polymorphism, biodiversity, chromosomes, chromosomal fusion/fission, cryptic species, cytogenetics, DNA barcoding, incomplete lineage sorting, karyosystematics, mitochondrial introgression, nomenclature, species identification, taxonomy

## Abstract

DNA barcoding has been suggested as a universal tool for molecular species identification; however, it cannot be applied in cases when morphologically similar species share their DNA barcodes due to the common ancestry or mitochondrial introgression. Here we analyze the karyotype of Polyommatus (Agrodiaetus) morgani (Le Cerf, 1909) from the region of its type locality in the southern Zagros Mountains in Iran, provide first chromosomal evidence for P. (A.) antidolus (Rebel, 1901) in Iran and demonstrate that these two species can be easily identified through analysis of their karyotypes whereas they share their mitochondrial barcodes.

## Introduction

Cryptic species, morphologically indistinguishable or highly similar biological entities, represent a substantial portion of plant and animal diversity, and therefore the search for these species is important for taxonomic, ecological and evolutionary studies (Beheregaray and Caccone 2007, Pfenninger and Schwenk 2007, Dincă et al. 2013, Vodă et al. 2015). Cryptic species can usually be identified through analysis of molecular markers (Vodă et al. 2015), e.g. through analysis of the so-called DNA barcodes, short genetic sequences from a standard part of the genome (Hebert et al. 2003). However, the use of the standard DNA barcodes such as short fragments of the mitochondrial gene *COI* and the non-coding nuclear sequence, *internal transcribed spacer 2* (*ITS2*), is sometimes insufficient to distinguish between evolutionarily young sister species, either because they can be weakly differentiated regarding these markers or because they are too polymorphic (Avise 2000, Lukhtanov et al. 2015a, b, 2016). The absence of lineage sorting among species can often pose a problem for the use of molecular markers in rapidly evolving taxa because the time to coalescence for alleles within lineages can be greater than the time required for speciation (Avise 2000, Kandul et al. 2004). Chromosomal characters in many groups can evolve more rapidly (Lukhtanov 2015, Vershinina and Lukhtanov 2017), and because they are often present as fixed differences, these characters could serve as applicable markers for recently evolved taxa (King 1993, Dobigny et al. 2005, Lukhtanov et al. 2015a, Vishnevskaya et al. 2016).

**Figure 1.**
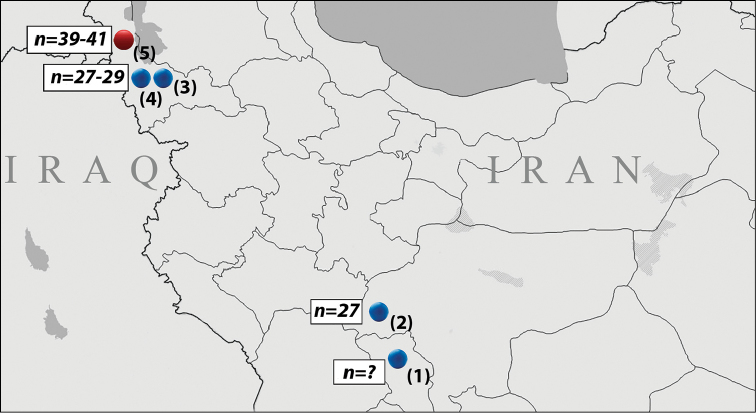
Map of Iran showing the type locality of Polyommatus (Agrodiaetus) morgani and the localities of the analyzed specimens of P. (A.) morgani and P. (A.) antidolus. **1** type locality of P. (A.) morgani, “Deh-Tcheshma” (Deh Cheshme near Farsan, Chaharmahal and Bakhtiari Province) **2** P. (A.) morgani, n=27, vic. Sibak, Esfahan Province **3** P. (A.) morgani, n=27-29, 25 km E of Mahabad, W. Azerbaijan Province **4** P. (A.) morgani, n=27-29, 15 km W of Mahabad, W. Azerbaijan Province **5** P. (A.) antidolus, n=39-41, Seir, 4 km S of Urmia, W. Azerbaijan Province.

**Figure 2.**
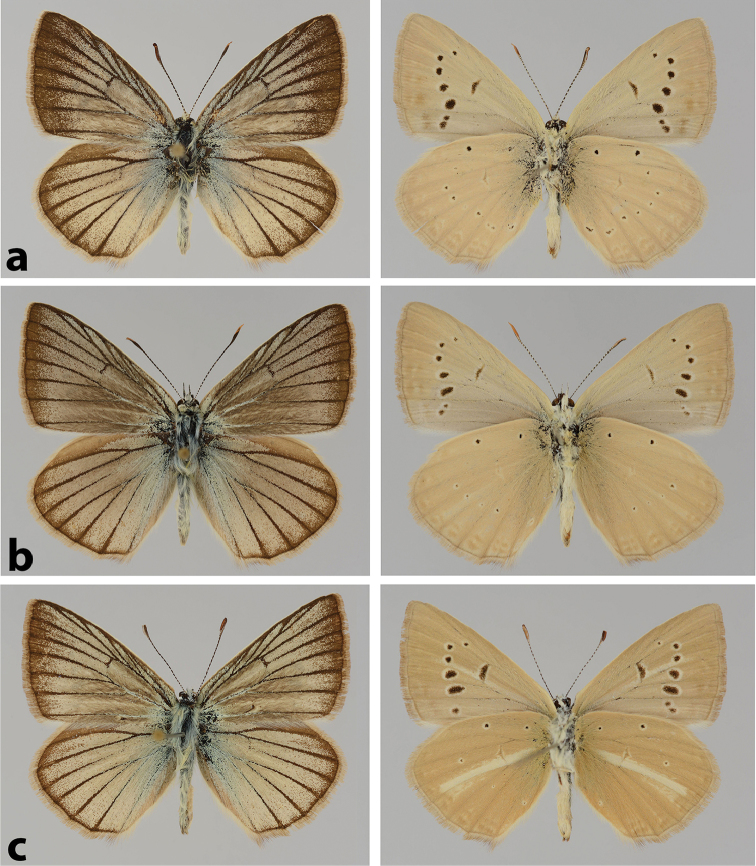
Male wing pattern of P. (A.) morgani, P. (A.) antidolus and P. (A.) kurdistanicus. **a** P. (A.) morgani, Iran, Kordestan Province, Senandaj, 1800 m, 20 July 2000, leg. P. Hofmann **b** P. (A.) antidolus Turkey, Hakkari Province, Ogul-Tal, 1500–1900 m, 1 August 1984, leg. Schurian **c** P. (A.) kurdistanicus Turkey, Van Province, 10 km S of Van, 1900–2100 m, 10 August 1978, leg. Görgner.

Polyommatus (Agrodiaetus) antidolus (Rebel, 1901), P. (A.) kurdistanicus (Forster, 1961) and P. (A.) morgani (Le Cerf, 1909), a complex of three closely related allopatric species distributed in east Turkey as well as in west and central Iran (Fig. 1) (Eckweiler and Bozano 2016), represent a good example of such situation. Despite morphological similarity (Fig. 2) and identity of *COI* barcodes in the majority of the studied populations (see Table 2 and sequences published in Wiemers 2003, Wiemers and Fiedler 2007, Kandul et al. 2004, 2007, Lukhtanov et al. 2015b and see Lukhtanov et al. 2015b for the exceptions), they can be easily identified by their chromosome numbers. Haploid chromosome numbers (n) were found to be n=25-27 in P. (A.) morgani, n=39-42 in P. (A.) antidolus and n=61-62 in P. (A.) kurdistanicus (de Lesse 1960, 1961, Lukhtanov et al. 1998, 2005, 2015b). However, the karyotype has never been studied in Iranian populations from the southern and northern Zagros Mountains including the region of the type locality of P. (A.) morgani (locality 1 in Fig. 1), and this negatively affects the identification and taxonomic interpretation of all known populations. Here we provide first chromosomal data for populations of the complex from the southern and northern Zagros Mountains.

## Material and methods

**Figure 3.**
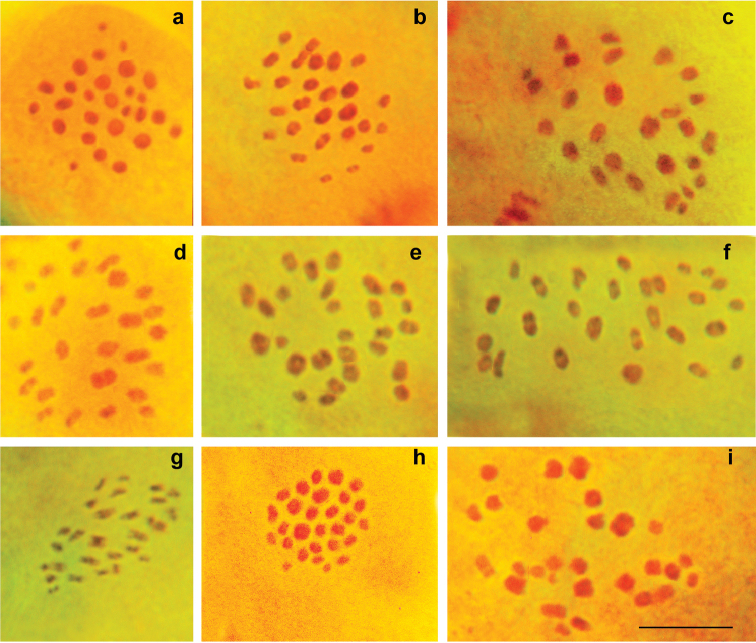
Karyotype of P. (A.) morgani **a** Q060, MI, n=27 **b** Q055, MI, n=27 **c** Q170, prometaphase I, n=27 **d** Q171, MI, n=27 **e** Q197, prometaphase I, n=27 **f** Q196, MI, n=27 **g** Q196, MII, n=27 **h** Q196, MII, n=28 **i** Q196, MII, n=28. Bar = 10 μ.

**Figure 4.**
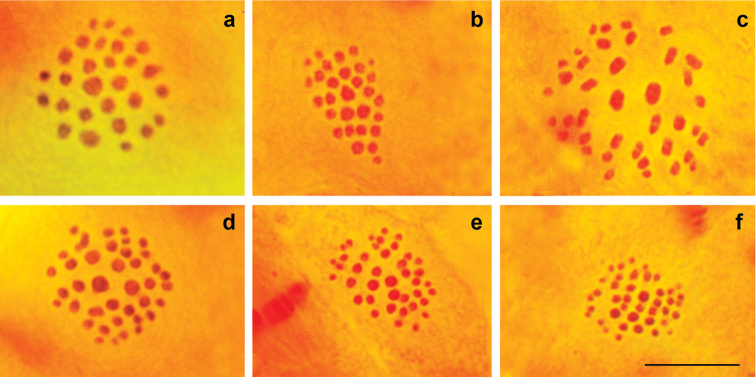
Karyotypes of P. (A.) morgani and P. (A.) antidolus. **a** P. (A.) morgani, Q150, MII, n=28 **b** P. (A.) morgani, Q150, MII, n=29 **c** P. (A.) antidolus, Q239, MI, n=39, squash preparation **d** P. (A.) antidolus, Q239, MI, n=39, intact metaphase plate **e** P. (A.) antidolus, Q237, MII, n=40 **f** P. (A.) antidolus, Q237, MII, n=41. Scale bar = 10 μ.

The butterflies were collected in 2016 in north-west and central Iran: in a mountain valley between Fereydunshahr and Sibak (locality 2), in the vicinity of Darman (25 km E of Mahabad) (locality 3), in the vicinity of Khalifen (15 km W of Mahabad) (locality 4) and in Seir (near Urmia) (locality 5) (Fig. 1). We also included sequences of karyotyped P. (A.) kurdistanicus and P. (A.) antidolus specimens available from GenBank (Wiemers 2003, Lukhtanov et al. 2005) in our analysis. A complete list of specimens included in this study and information about sampling localities are given in Table 1. Karyotypes (Figs 3 and 4) and *COI*-barcodes (Table 1 and 2) were analyzed using approaches described previously (Lukhtanov et al. 2014, Przybyłowicz et al. 2014). We use the following abbreviations: MI for metaphase I of meiosis and MII for metaphase II of meiosis. Divergences between *COI* sequences were computed using MEGA6 software (Tamura et al. 2013).

**Table 1.** List of studied material (17 specimens). Asterisks indicate unsequenced specimens. Collectors: E. Pazhenkova (EP), N. Shapoval (NS), V. Lukhtanov (VL).

Field Code	GenBank number	Taxon	Chromosome number (n)	Locality	Altitude	Date	Collectors/ References
Q055*		*morgani* ♂	n=27	Iran, Esfahan Prov., Sibak (N32°55'; E50°04')	2700 m	02.08.2017	EP, NS, VL
Q060*		*morgani* ♂	n=27	Iran, Esfahan Prov., Sibak (N32°55'; E50°04')	2700 m	02.08.2017	EP, NS, VL
Q150	MG457163	*morgani* ♂	n=28-29	Iran, W. Azerbaijan Prov., vic. Darman, 25 km E of Mahabad (N36°45'; E45°52')	1900–2000 m	10.08.2017	EP, NS, VL
Q170	MG457164	*morgani* ♂	n=27	Iran, W. Azerbaijan Prov., vic. Darman, 25 km E of Mahabad (N36°45'; E45°52')	1900–2000 m	10.08.2017	EP, NS, VL
Q171	MG457165	*morgani* ♂	n=27	Iran, W. Azerbaijan Prov., vic. Darman, 25 km E of Mahabad (N36°45'; E45°52')	1900–2000 m	10.08.2017	EP, NS, VL
Q181	MG457166	*morgani* ♂	n=28	Iran, W. Azerbaijan Prov., vic. Darman, 25 km E of Mahabad (N36°45'; E45°52')	1900–2000 m	10.08.2017	EP, NS, VL
Q196	MG457167	*morgani* ♂	n=27-28	Iran, W. Azerbaijan Prov., vic. Khalifen, 15 km W of Mahabad (N36°45'; E45°32')	2100–2200 m	11.08.2017	EP, NS, VL
Q197	MG457168	*morgani* ♂	n=27	Iran, W. Azerbaijan Prov., vic. Khalifen, 15 km W of Mahabad (N36°45'; E45°32')	2100–2200 m	11.08.2017	EP, NS, VL
Q198	MG457169	*morgani* ♂	n=28-29	Iran, W. Azerbaijan Prov., vic. Khalifen, 15 km W of Mahabad (N36°45'; E45°32')	2100–2200 m	11.08.2017	EP, NS, VL
Q237	MG457170	*antidolus* ♂	n=40-41	Iran, W. Azerbaijan Prov., vic. Seir, Urmia (N37°28'; E45°02')	1750 m	14.08.2017	EP, NS, VL
Q238	MG457171	*antidolus* ♂	n=39-40	Iran, W. Azerbaijan Prov., vic. Seir, Urmia (N37°28'; E45°02')	1750 m	14.08.2017	EP, NS, VL
Q239	MG457172	*antidolus* ♂	n=39	Iran, W. Azerbaijan Prov., vic. Seir, Urmia (N37°28'; E45°02')	1750 m	14.08.2017	EP, NS, VL
	AY557093	*antidolus* ♂	n=42	Turkey, Hakkari Prov., Dez Çay	1500 m	22.07.1999	Wiemers 2003
	AY557095	*antidolus* ♂	n=ca44	Turkey, Hakkari Prov., Haruna Geçidi, SE Yüksekova	2000 m	21.07.1999	Wiemers 2003
	AY557108	*kurdistanicus* ♂	n=ca>55	Turkey, Van Prov., Erek Dagi	2200 m	25.07.1999	Wiemers 2003
	AY557074	*kurdistanicus* ♂	n=ca54-56	Turkey, Van Prov., Çatak	1600–1900 m	25.07.1999	Wiemers 2003
	AY496762	*kurdistanicus* ♂	n=62	Turkey, Van Prov., Çatak		July 2001	Lukhtanov et al. 2005

**Table 2.** Divergence between *COI* sequences. The numbers of base differences per site between sequences are shown. The shared barcodes (uncorrected *COI p*-distance = 0) are shown in bold.

	1	2	3	4	5	6	7	8	9	10	11	12	13	14	15
(1) AY557095 *antidolus*															
(2) AY557089 *antidolus*	0.0015														
(3) AY557108 *kurdistanicus*	0.0015	**0**													
(4) AY557074 *kurdistanicus*	0.0015	**0**	**0**												
(5) AY496762 *kurdistanicus*	0.0015	**0**	**0**	**0**											
(6) Q150 *morgani*	0.0015	**0**	**0**	**0**	**0**										
(7) Q170 *morgani*	0.0029	**0**	**0**	**0**	**0**	**0**									
(8) Q171 *morgani*	0.0029	0.0015	0.0015	0.0015	0.0015	0.0015	0.0015								
(9) Q181 *morgani*	0.0015	**0**	**0**	**0**	**0**	**0**	**0**	0.0015							
(10) Q196 *morgani*	0.0015	**0**	**0**	**0**	**0**	**0**	**0**	0.0015	**0**						
(11) Q197 *morgani*	0.0015	**0**	**0**	**0**	**0**	**0**	**0**	0.0015	**0**	**0**					
(12) Q198 *morgani*	0.0015	**0**	**0**	**0**	**0**	**0**	**0**	0.0015	**0**	**0**	**0**				
(13) Q237 *antidolus*	0.0015	**0**	**0**	**0**	**0**	**0**	**0**	0.0015	**0**	**0**	**0**	**0**			
(14) Q238 *antidolus*	0.0015	**0**	**0**	**0**	**0**	**0**	**0**	0.0015	**0**	**0**	**0**	**0**	**0**		
(15) Q239 *antidolus*	0.0015	**0**	**0**	**0**	**0**	**0**	**0**	0.0015	**0**	**0**	**0**	**0**	**0**	**0**	

## Results and discussion

In order to investigate the topotypical population of P. (A.) morgani, we first searched for it in its exact type locality in “Deh Tcheshma” (mountain area near the village Deh Cheshme, close to the city Farsan, Chaharmahal and Bakhtiari Province, Iran) (locality 1 in Fig. 1). Unfortunately, we were unable either to find it there or to locate a biotope suitable for butterflies of the P. (A.) antidolus - P. (A.) kurdistanicus - P. (A.) morgani complex. In our opinion, P. (A.) morgani is extinct in its type locality, probably due to climate change and aridification during the last 100 years. Fortunately, we were able to find typical P. (A.) morgani in a small, relatively humid mountain valley between Fereydunshahr and Sibak, 90 km NW of Farsan (N32°55; E50°04’, Esfahan Province, Iran) (locality 2 in Fig. 1). In two studied specimens from the latter locality, at the MI stage, the haploid chromosome number n = 27 was found (Figs 3a, b). The meiotic karyotype was strongly asymmetric, with a group of larger bivalents (from 6 to 10 in different cells) and a group of smaller bivalents (from 17 to 21 in different cells). The number of bivalents that were classified as “larger” and “smaller” was variable, most likely depending on the bivalent orientation. However, in some metaphase plates, the distinction between the larger and smaller bivalents was unclear, and the bivalents gradually decreased in size, with the largest bivalent approximately 10 times larger than the smallest one. Thus, the results obtained confirm the previous taxonomic interpretations (de Lesse 1960, 1961, Carbonell 2003, Lukhtanov et al. 1998, 2005, 2015b, Eckweiler and Bozano 2016) that considered the populations with n=25–27 as P. (A.) morgani.

Tshikolovets et al. (2014) and Eckweiler and Bozano (2016) identified the population of the P. (A.) antidolus - P. (A.) kurdistanicus - P. (A.) morgani complex from the vicinity of Mahabad (West Azerbaijan Province) (localities 3 and 4 in Fig. 1) as P. (A.) antidolus; however, they did not provide any chromosomal data to confirm this conclusion. We analyzed seven specimens from two localities close to Mahabad (localities 3 and 4 in Fig. 1). At the prometaphase I, MI and MII stages, n=27 was determined as the basic number in four specimens (Fig. 3c–g), not n=39-42 as expected for P. (A.) antidolus. The number of elements within the karyotype was unstable, varying from n=27 to n=29, most likely due to the presence of two chromosomal fusions/fissions (Figs 3h, i, 4a, b). With respect to the karyotype structure (size and proportion of larger vs. smaller chromosomal elements) the specimens from Mahabad were indistinguishable from the typical P. (A.) morgani described above. The chromosome numbers n=28 and n=29 were not previously reported for P. (A.) morgani (de Lesse 1960, 1961, Lukhtanov et al. 1998, 2005, 2015b). However, since there is no fixed chromosomal difference between the populations from Sibak and Mahabad, we do not see the need for a description of a new taxon from Mahabad, and therefore identify the populations from Mahabad as P. (A.) morgani.

Finally, in three specimens collected in Seir (near Urmia, locality 5 in Fig. 1) at the MI/MII stages, we found that the number of chromosomal elements varied from 39 to 41. The chromosomes ranged in size from very small to large (Fig. 4c–f). This karyotype (n=39-41) seems to be identical to that found in P. (A.) antidolus in the neighboring Province Hakkari in south-east Turkey, thus providing first chromosomal evidence for P. (A.) antidolus in Iran.
